# High-throughput fermentation screening for the yeast *Yarrowia lipolytica* with real-time monitoring of biomass and lipid production

**DOI:** 10.1186/s12934-016-0546-z

**Published:** 2016-08-23

**Authors:** Alexandre Back, Tristan Rossignol, François Krier, Jean-Marc Nicaud, Pascal Dhulster

**Affiliations:** 1 Univ. Lille, INRA, ISA, Univ. Artois, Univ. Littoral Côte d’Opale, EA 7394-ICV- Institut Charles Viollette, F-59000 Lille, France; 2 Micalis Institute, INRA, AgroParisTech, Université Paris-Saclay, 78350 Jouy-En-Josas, France

**Keywords:** *Yarrowia lipolytica*, Screening, High throughput, Lipid production

## Abstract

**Background:**

Because the model yeast *Yarrowia lipolytica* can synthesize and store lipids in quantities up to 20 % of its dry weight, it is a promising microorganism for oil production at an industrial scale. Typically, optimization of the lipid production process is performed in the laboratory and later scaled up for industrial production. However, the scale-up process can be complicated by genetic modifications that are optimized for one set of growing conditions can confer a less-than-optimal phenotype in a different environment. To address this issue, small cultivation systems have been developed that mimic the conditions in benchtop bioreactors. In this work, we used one such microbioreactor system, the BioLector, to develop high-throughput fermentation procedures that optimize growth and lipid accumulation in *Y. lipolytica*. Using this system, we were able to monitor lipid and biomass production in real time throughout the culture duration.

**Results:**

The BioLector can monitor the growth of *Y. lipolytica* in real time by evaluating scattered light; this produced accurate measurements until cultures reached an equivalent of OD_600nm_ = 115 and a cell dry weight of 100 g L^−1^. In addition, a lipid-specific fluorescent probe was applied which reliably monitored lipid production up to a concentration of 12 g L^−1^. Through screening various growing conditions, we determined that a carbon/nitrogen ratio of 35 was the most efficient for lipid production. Further screening showed that ammonium chloride and glycerol were the most valuable nitrogen and carbon sources, respectively, for growth and lipid production. Moreover, a carbon concentration above 1 M appeared to impair growth and lipid accumulation. Finally, we used these optimized conditions to screen engineered strains of *Y. lipolytica* with high lipid-accumulation capability. The growth and lipid content of the strains cultivated in the BioLector were compared to those grown in benchtop bioreactors.

**Conclusion:**

To our knowledge, this is the first time that the BioLector has been used to track lipid production in real time and to monitor the growth of *Y. lipolytica*. The present study also showed the efficacy of the BioLector in screening growing conditions and engineered strains prior to scale-up. The method described here could be applied to other oleaginous microorganisms.

## Background

The depletion of petroleum reserves and the increase in petroleum by-product consumption have motivated researchers to search for alternatives to fossil-derived oil [1]. In this context, microbial oil appears to be a promising alternative, particularly the oil produced by oleaginous yeasts, as they can be easily cultivated and genetically enhanced [1–7]. In addition, as the lipids of oleaginous yeasts are similar to fats from plant seeds, they can be considered an alternative to agricultural oil sources as well [8].

Although *Yarrowia lipolytica* accumulates lipids at a lower level than other oleaginous yeasts (i.e. *Rhodotorula glutinis*, *Trichosporon pullulans*, *Cryptococcus albidus*, and *Rhodosporidium toruloides*), it remains a promising microorganism for study because its genome has been fully sequenced [9] and annotated, and efficient genetic tools are available for its study [10–12]. In all oleaginous yeasts, nitrogen limitation is commonly used to induce the accumulation of intracellular lipids [2]. During the growth phase, both nitrogen and carbon are used for the synthesis of proteins and nucleic acids necessary for growth, while carbon is also used for the synthesis of carbohydrates and lipids. When nitrogen becomes limiting, growth slows down and the carbon flux is preferentially used for lipid synthesis [2]. In addition to the carbon-to-nitrogen (C/N) ratio, factors such as pH, dissolved oxygen concentration, and substrate identity can also influence lipid accumulation [13–15].

In *Y. lipolytica*, microbial engineering has been used to improve lipid accumulation. In yeast cells, lipids are stored in particles called lipid bodies, mainly in the form of triacylglycerol (TAG). A limiting factor that affects lipid production is the synthesis of TAG from diacylglycerol (DAG) by DAG acyltransferases (DGATs). Recently, DGATs were identified and characterized in *Y. lipolytica* [16, 17], and it was shown that the overexpression of DGATs, and in particular, *YlDGA2*, can increase lipid accumulation by a factor of 1.7 [17, 18].

The conditions for the fermentation process and metabolic engineering have both been mainly optimized for lipid production at a laboratory scale in shake flasks. However, the optimal growth conditions and genetic modifications for a laboratory setting are not necessarily the best-adapted for an industrial setting, and even slight deviations from the optimum can seriously affect industrial productivity. In particular, an erroneous assessment of growth and production conditions in the upstream development phase could greatly impair the economic viability of the bioprocess [19]. For this reason, high-throughput screening techniques and small-scale cultivation systems have been developed to accelerate process development and decrease costs by avoiding scale-up issues [20, 21]. These techniques are intended to help with the screening of strains, growing conditions, and substrates for industrial fermentation and to enable the detection of growth inhibitors in crude extracts which may impair the process. For example, microtiter plate readers are useful tools to efficiently screen multiple growing conditions. In addition, fluorescent probes (e.g., Bodipy) can be used to specifically measure lipid accumulation in real time [22]. However, the utility of microtiter plates is limited due to their small working volume, which means that experimental data can only be evaluated by endpoint assays. To address this problem, scalable systems have been developed in which growth, pH, and dissolved oxygen can be monitored in real time [20, 23–26]. In particular, the BioLector microtiter plate reader (from m2p labs, Baesweiler, Germany) allows 48 parallel fermentations to take place in one run. It mimics the oxygen transfer rate and growth obtained in bioreactors but also enables the monitoring of fluorescence signals during fermentation. Fermentations conducted in the BioLector have led to protein production similar to that obtained in a 1.4 L bioreactor [19, 22]. Using the BioLector, it is possible to have the best of both worlds: like a bioreactor, it has the ability to track growth conditions in real time, and like standard microtiter plates, it enables rapid, high-throughput cultivation with online fluorescence monitoring. This valuable tool can be used to determine the optimal conditions for TAG production, to screen libraries of strains or mutants for differences in activity, and to evaluate various substrates to determine the optimal growth conditions for lipid production [26].

Thus far, the BioLector has been successfully used with the model bacterium *Escherichia coli* and the yeast *Hansenula polymorpha* to scale up from microtiter plates to a bioreactor and to screen optimal growing conditions; it has also been used for the production of the green fluorescent protein [19, 21, 22]. The aim of this work was to evaluate, in real time, both growth and lipid accumulation of *Y.* *lipolytica* in a BioLector microfermentor system and to use this information to establish methodologies for high-throughput fermentation screening of various strains and substrates, with an eye towards optimizing lipid production. Our goal was also to validate the BioLector as an instrument that provides useful information to support decision-making in fermentation scale-up.

## Methods

### Yeast strains and culture media

The strains of *Y. lipolytica* used in this study are listed in Table 1. They are derived from the auxotrophic mutant Po1d (*ura*^−^, *leu*^−^), which is itself derived from the wild-type strain W29 (ATCC 20460). Strain JMY3675 was obtained by transformation of JMY330 with an expression cassette that contained both the *SUC2* gene under the pTEF promoter and the *LEU2ex* marker (see Reference [28] for the experimental procedure). Inocula were prepared via culturing at 28 °C and 160 rpm in rich YPD medium that contained 5 g L^−1^ of yeast extract, 10 g L^−1^ of peptone, and 20 g L^−1^ of glucose. Two types of media were used for fermentation: synthetic medium and rich medium. Unless otherwise indicated, all reagents used in medium preparation were obtained from Sigma-Aldrich (St. Louis, USA). The synthetic medium was composed of 0.85 g L^−1^ of yeast nitrogen base without amino acids and ammonium sulfate (YNB; BD Difco, Franklin Lakes, United States), 50 mM phosphate buffer (pH 6.8), ammonium chloride (Nc) or ammonium sulfate (Ns) and glycerol (G). The glycerol was mixed with one of the ammonium sources to reach a C/N molar ratio of either 25 or 35. The rich medium was composed of 5 g L^−1^ of yeast extract (Y) and 10 g L^−1^ of peptone (P) and supplemented with glucose (D), glycerol (G), or sucrose (S) in order to obtain C/N molar ratios of 3.5, 15, 25, 35, or 45. In the following sections, the concentration (g L^−1^) of each component of the medium is indicated by the number after the letter corresponding to the component in question, for instance Y10 stands for 10 g L^−1^ of yeast extract.

**Table 1:** *Y. lipolytica* strains used in this study

Strain	Genotype	References
W29 (ATCC 20460)	MATA, *wild*-*type*	[46]
Po1d	MATA *ura3*-*302*:*pXPR2::SUC2 leu2*-*270 xpr2*-*322*	[49]
JMY330	Po1d Ura+	[47]
JMY2900	Po1d Ura+, Leu+	[19]
JMY3675	JMY330+ LEU2ex-pTEF-SUC2	Unpublished
JMY1631	Po1d, *Δdga1*, *Δlro1*, *Δare1*::*URA3, Δdga2*::*LEU2*	[18]
JMY3400	Po1d, *Δlro1*, *Δdga1*, *Δdga2*, *Δare2*, *URA3ex*-pTEF-*DGA2*, Leu+	[19]
JMY3357	Po1d, *Δlro1, Δdga1, Δdga2, Δare2, URA3ex*-pTEF-*DGA2*, *LEU2*-pTEF-*DGA2*	[18]
JMY3580	Po1d, *Δlro1, Δdga1, Δdga2, Δare2,* 2xpTEF-*DGA2*, *URA3ex*-pTEF-*DGA2*, *LEU2*-pTEF-*DGA2*	[18]

### Calibration of biomass and lipid accumulation detection

Biomass calibration was performed following the method described by Kensy et al. [23]. Briefly, *Y.* *lipolytica* JMY3675 was cultivated in Y5P10D20 rich medium that was supplemented with 1 µg/mL of Bodipy (ex: 493 nm/em: 503 nm, ThermoFisher Scientific, Illkirch-Graffenstaden, France). The strain was cultured at 28 °C and 160 rpm (4 mm shaking diameter; KS 130 control orbital shaker, IKA, Staufen, Germany) for 48 h in two 1 L shake flasks. The cultures were harvested and concentrated to 25× by centrifugation (4000*g*; centrifuge 5804R, Eppendorf, Hamburg, Germany) in physiological water (9 g L^−1^ NaCl). The concentrated culture was diluted in physiological water and the cell dry weight (CDW), scattered light intensity, fluorescence (ex: 488 nm/em: 520 nm, Gain: 10), and OD_600nm_ of each dilution were determined. The lipid content of each dilution was also analyzed by gas chromatography as described below in order to evaluate the correlation between fluorescence and the concentration of intracellular lipids.

### BioLector growing conditions

*Yarrowia lipolytica* growth in the BioLector (mp2-labs, Baesweiler, Germany) was performed in rich medium and in synthetic medium in triplicate on one plate. Media were complemented with 0.2 M KI to avoid nonspecific fluorescence [26, 28]. Cells were precultivated for 48 h in Y5P10D20 medium, washed, and inoculated at an OD_600nm_ of 1 in each well. The experiments were performed at 28 °C under constant agitation (1200 rpm, shaking diameter = 3 mm, orbital) in 48-well FlowerPlates (mp2-labs, Baesweiler, Germany) with a working volume of 800 µL. Growth was monitored by measuring scattered light intensities (ex: 620 nm/em: -, Gain: 10). In each well, a final concentration of 1 µg/mL of either Bodipy (Thermo Fisher Scientific, Waltham, United States) or Nile red (Sigma-Aldrich, St. Louis, United States) was added to evaluate the lipid concentration using either the green fluorescent protein optical filter (ex: 488 nm/em: 520 nm, Gain: 80) or the Nile red filter (ex: 589 nm/em: 671 nm, Gain: 80), respectively.

### Bioreactor conditions

Batch cultivation of *Y. lipolytica* was performed in a 500 mL bioreactor (Applikon Biotechnology B.V., Delft, Netherland) with a working volume of 200 mL in synthetic medium with an initial C/N ratio of 35 (i.e. 0.333 M of glycerol and 0.029 M of NH_4_Cl). The medium was inoculated to an OD_600nm_ of 1 using a 48 h preculture. The temperature was held constant at 28 °C, the agitation at 1000 rpm, and the dissolved oxygen at 30 % saturation by injection of 0.2 to 2 VVM of air. Foam was prevented by the addition of 0.01 % (V/V) of an antifoam agent (Rhodorsil; Rhodia, La Defense, France) and by placing a Rushton turbine at the medium/air interface.

### Lipid analysis by gas chromatography

Fatty acids from lyophilized cells were converted to their fatty acid methyl ester counterparts using the method described in [29] and [30]. Fatty acid methyl esters were analyzed on a Varian 3900 gas chromatograph that was equipped with a flame ionization detector and a Varian Factor Four vf-23 ms column, for which the specifications at 260 °C were 3 pA (30 m, 0.25 mm, 0.25 μm). Fatty acids were identified by comparing their chromatograms to those of commercial fatty acid methyl ester standards (FAME32; Supelco) and quantified using the internal standard method with 100 mg of commercial C12:0 (Sigma-Aldrich, St. Louis, United States).

## Results and discussion

### Detection of *Yarrowia lipolytica* biomass and intracellular lipids via analysis of scattered light and fluorescence

**Fig. 1:**
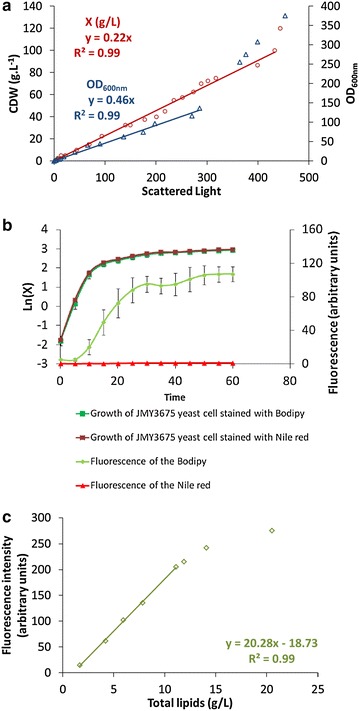
Evaluation of *Y. lipolytica* biomass monitoring with scattered light and lipid accumulation with fluorescent dyes. **a** The limits of biomass monitoring using scattered light were evaluated by comparing scattered light measurements with those of CDW or OD_600nm_. **b** The ability of two probes (*Nile red* and Bodipy) to measure total lipid production was evaluated using 1 µg/mL of both probes in a defined medium that contained 0.85 g L^−1^ of YNB, 1.5 g L^−1^ of NH_4_Cl, and 30.6 g L^−1^ of glycerol. **c** In each well, 1 µg/mL of the intracellular lipophilic probe Bodipy (ex: 486 nm/em: 508 nm) was added and the limit of intracellular lipid monitoring with fluorescence detection was evaluated. Scattered light (ex: 620 nm/em: -, Gain: 10), Bodipy fluorescence (ex: 488 nm/em: 520 nm, Gain: 80), and *Nile red fluorescence* (ex: 589 nm/em: 671 nm, Gain: 80) were measured using a 48-well flowerplate with 800 µL filling volume at 28 °C and 1200 rpm (shaking diameter: 3 mm)

High-throughput growth screening is usually conducted in standard 96-well plates; however, these have known disadvantages with regard to growth because oxygen transfer is limited. In addition, microtiter plate readers typically monitor growth by determining the OD_600nm_, but OD_600nm_ measurements are notorious for consistently underestimating biomass at high concentrations. The BioLector addresses both of these issues: to improve oxygen transfer, it uses flowerplates specifically designed to mimic the strong agitation present in bioreactors, and to monitor growth, it measures scattered light, which has been demonstrated to generate higher measurements of biomass concentration than OD_600nm_ does [21, 23, 31]. As a first step, we established the limits of online biomass detection by scattered light as well as the online detection of lipid concentration by Bodipy fluorescence (see ‘‘Methods’’ section). Strain JMY3675, which expresses an optimized invertase expression cassette, was used to evaluate these parameters. This expression cassette contains the *Saccharomyces cerevisiae SUC2* gene under the control of the strong promoter pTEF, and has been shown to confer efficient growth both in sucrose and in cost-effective industrial sucrose-based substrates like molasses [18, 27, 32, 33]. Moreover, in Y5P10D15 (C/N = 3.5) medium, strain JMY3675 did not differ significantly from the W29 wild-type strain in growth or lipid accumulation (data not shown), showing that it was a suitable candidate for the calibration of the BioLector. For calibration, JMY3675 was cultured in shake flasks and then concentrated in physiological water. Dilutions of this culture were then used to estimate the correlations between scattered light, CDW, and OD_600nm_, as well as the correlation between fluorescence and intracellular lipid concentration as determined by gas chromatography (GC). The correlation between scattered light and CDW, as shown in Fig. 1, was linear up to a CDW concentration of 100 g L^−1^. Below this concentration, one unit of scattered light was equal to 0.22 g L^−1^ of CDW. Likewise, scattered light measurements displayed a linear correlation with those of OD_600nm_ (Fig. 1a), in which one unit of scattered light was equal to 0.46 units of OD_600nm_. The limit of the OD_600nm_ monitoring via scattered light was 115, which is equivalent to a CDW of 58 g L^−1^ (1 unit of OD_600nm_ is equal to 0.5 g L^−1^ of CDW).The limit of CDW monitoring by scattered light was 100 g L^−1^, thus, it is possible to track higher levels of CDW using scattered light than with OD_600nm_. Two fluorescent probes, Nile red and Bodipy, were evaluated for use in monitoring the production of intracellular lipids (Fig. 1b). These two probes are commonly used to stain intracellular lipids for the purpose of quantification [28, 34–36]. No differences in growth were observed between cells stained with Nile red or those stained with Bodipy, but Bodipy was the better performer of the two in terms of quantifying lipid production by *Y.* *lipolytica* in our conditions (Fig. 1b). Surprisingly, we did not observe any fluorescence when Nile red was used. To our knowledge, previous studies that quantified lipid production using Nile red did so solely offline [35, 36], and our efforts represent the first attempts at online measurements. The lack of fluorescence observed here could be due to the fact that Nile red appears to be less photo-stable than Bodipy [37]. Unfortunately, it is difficult to compare the performance of the two probes due to the lack of data concerning Bodipy and because the marking response appears to be strictly species-dependent [37]. With Bodipy, a linear correlation was observed between fluorescence and intracellular lipid concentration up to 200 arbitrary fluorescent units (corresponding to 12 g L^−1^ of lipids) (Fig. 1c). Up to this point, the intracellular lipid concentration could be calculated using the following formula:

$${\text{Intracellualr}}\;{\text{lipids}}\;({\text{g}}/{\text{L}})\; = \;\frac{{{\text{Fluorescent}}\;{\text{units}}\; + \;18.73}}{{20.28}}$$

It must be noted, however, that the correlations described above depend on the device calibration and must be revised when the apparatus is recalibrated. Furthermore, although Bodipy fluorescence accurately correlated with lipid concentrations up to 12 g L^−1^, this threshold represents a significant limitation for the utility of this approach, as some *Y. lipolytica* strains are able to produce more than 25 g L^−1^ of lipids [3, 38]. To solve this problem, alternative methods using automation and microtiter plates might be developed, as in 36. However, as with all microtiter plate applications, such methods would only be suitable for endpoint sampling and thus would be complementary to the techniques described here.

### Influence of carbon source on growth and on intracellular lipid accumulation

To our knowledge, the BioLector has never before been used to monitor the growth of *Y. lipolytica* or to monitor lipid production using Bodipy. For a preliminary assessment of its utility for these purposes, then, we grew the first set of cultures in rich medium, as it is more efficient than defined medium for both biomass and lipid production. In oleaginous yeasts, both of these activities are regulated by the C/N ratio [2]. In order to stimulate lipid accumulation, JMY3675 was grown on rich media that contained glucose (YPD), glycerol (YPG), or sucrose (YPS) as the carbon substrate at four carbon concentrations: 2.2, 3.6, 5.1 and 6.5 M, which corresponded to C/N ratios of 15, 25, 35, and 45, respectively.

**Fig. 2:**
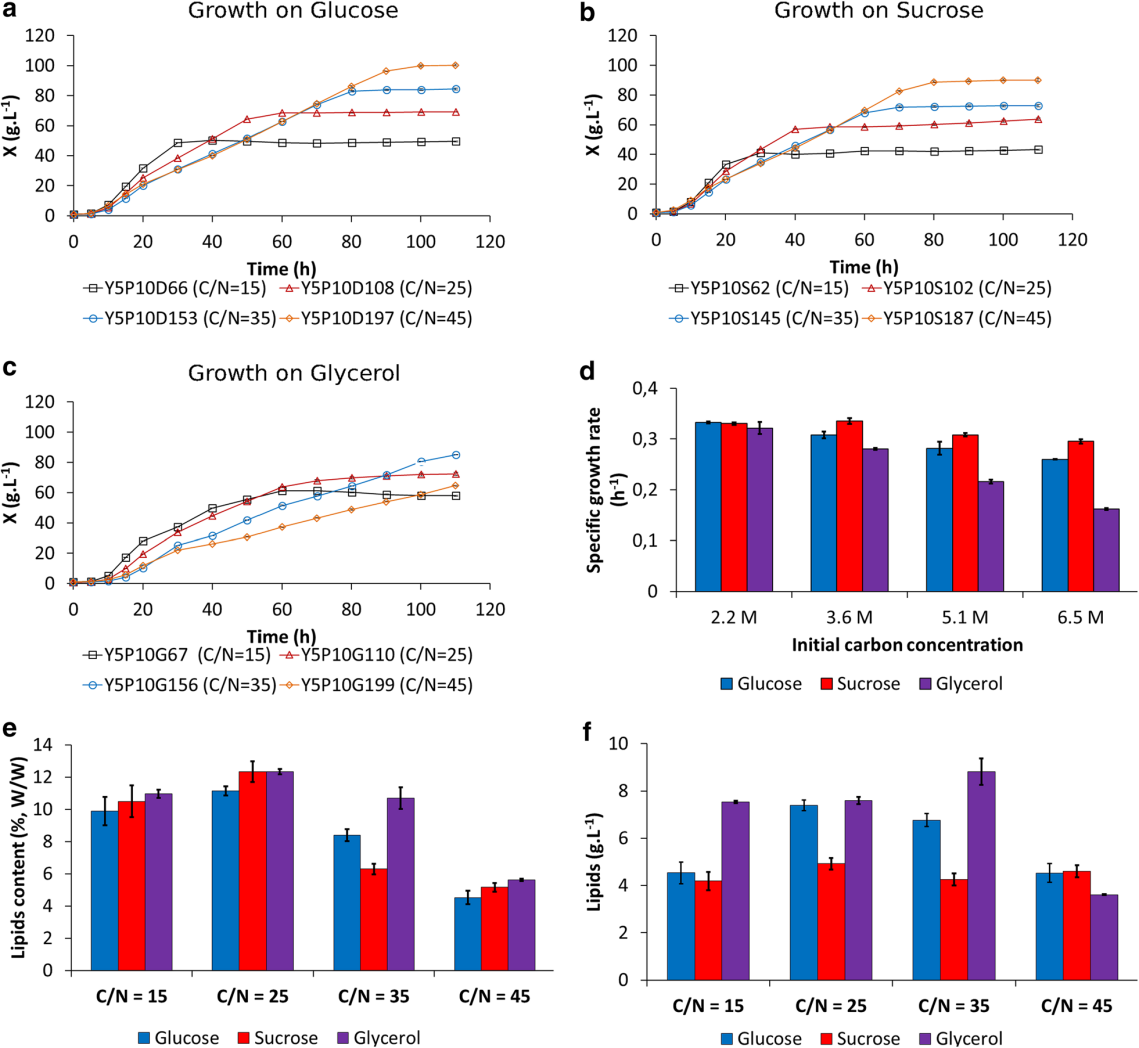
Growth of *Y. lipolytica* using different carbon sources in a rich medium. *Y. lipolytica* JMY3675 was cultured in rich medium that was supplemented with either glucose (D; Fig. 2a), sucrose (S; Fig. 2b), or glycerol (G; Fig. 2c). The growth of this strain was monitored in a 48-well flowerplate (filling volume: 800 µL, temperature: 28 °C, agitation: 1200 rpm) by measuring scattered light (ex: 620 nm/em: -, Gain: 10). In each medium, the sugars and glycerol were mixed with peptones and yeast extract to reach a C/N ratio of 15, 25, 35, or 45, as indicated in the legends. The figure was plotted by transforming units of scattered light into CDW (X in g L^−1^; *line*). **d** The specific growth rate of the strain was determined for each carbon source and initial carbon concentration. **e** Intracellular lipid yield and **f** total lipids at 110 h of culture were evaluated by measuring Bodipy fluorescence

Glucose appeared to be more efficient in producing biomass than glycerol or sucrose was (Fig. 2a–c) after 110 h of growth. With glucose- and sucrose-containing media, an increase in the C/N ratio resulted in increased biomass production. With glycerol, instead, the highest biomass production (86 g L^−1^) occurred at a C/N of 35 after 110 h of culture. However, due to the low specific growth rate on glycerol at a C/N of 45 (Fig. 2d), growth arrest was only obtained after 200 h of culture. At this time, the biomass of cultures with a C/N of 45 was higher (93 g L^−1^) than for a C/N of 35 (data not shown). The maximal specific growth rate (*µ*_max_) slightly decreased when the concentration of sucrose and glucose increased (Fig. 2d). More dramatically, the *µ*_max_ decreased from 0.32 h^−1^ to 0.16 h^−1^ between glycerol concentrations of 2.2 and 6.5 M (Fig. 2d). As described below (Fig. 4), high carbon concentration seemed to be deleterious for growth, probably because of increased osmotic pressure. In general, lipid accumulation in batch mode depends on the duration of the growth phase and on the initial C/N ratio [2]. Our results show that when the initial C/N ratio increased, a decrease of the maximal specific growth rate was observed.

A C/N ratio of 25 seemed to be slightly more efficient overall in promoting lipid accumulation, but these results were not significantly different from those obtained with a C/N of 35 (*t* test; *p*-value >0.05). A C/N ratio of 45 was the least efficient regardless of the carbon source (Fig. 2e). In oleaginous yeasts, higher C/N ratios have been reported to promote citric acid synthesis at the expense of lipid production. Citric acid is a precursor for lipid synthesis and it has been hypothesized that a key enzyme for lipid synthesis, ATP-citrate lyase, is inactivated by an excess of glucose [39]. In batch culture, Beopoulos and colleagues observed that a high initial C/N molar ratio (between 80 and 120) induced significant citric acid production and a low level of lipid accumulation in *Y. lipolytica* [2]. Similar results for C/N ratios above 100 were observed in batch cultures of *Y. lipolytica* performed with crude glycerol [40]. Despite this, other studies have reported that C/N ratios of 70 and 100 efficiently induce lipid accumulation in *Y. lipolytica* [41, 42]. In addition, Blazeck et al. showed that discrepancies in lipid accumulation in *Y.* *lipolytica* might be influenced by glucose concentration [3]. These differences may also be due to variation in growing conditions, such as aeration, pH, and medium components, which also influence lipid accumulation [2]. In our preliminary cultures, patterns of intracellular lipid accumulation appeared to resemble those of growth in glucose- and glycerol-based media (Fig. 2f). Instead, in sucrose media, lipid production remained constant regardless of the C/N ratio (Fig. 2f), probably because the efficient utilization of fructose was limited by hexokinase activity, as reported in 43.

The highest overall lipid production was obtained from cultures on glycerol-containing medium with a C/N ratio of 35 (Fig. 2f). Likewise, a C/N ratio of 35 resulted in higher biomass on this substrate than did a C/N ratio of 25 (Fig. 2a, d). We therefore decided to use these conditions (glycerol-based medium, C/N ratio of 35) for all following experiments.

### Influence of nitrogen on intracellular lipid accumulation

**Fig. 3:**
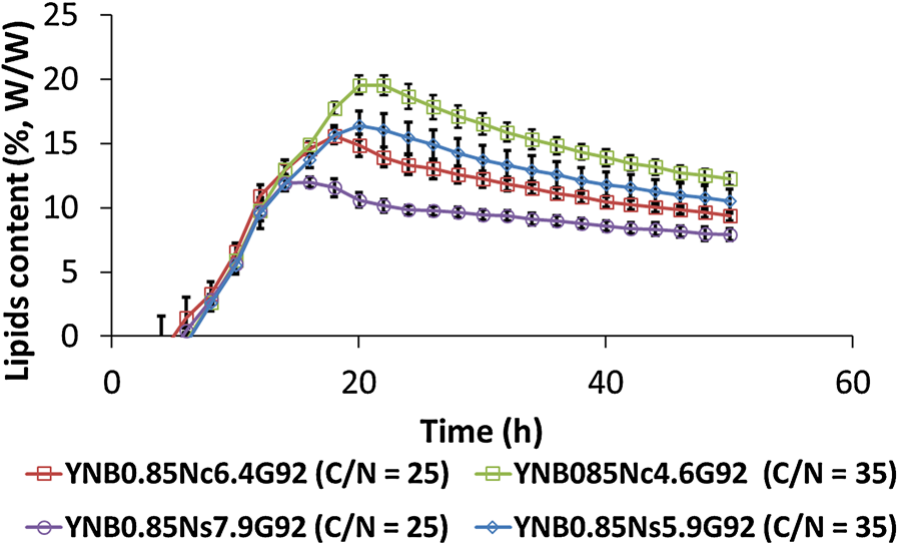
Growth of *Y. lipolytica* using different nitrogen sources in a synthetic medium. The yeast strain *Y. lipolytica* JMY3675 was cultured in a synthetic medium using glycerol as the carbon source and either ammonium chloride or ammonium sulfate as the nitrogen source. Nitrogen sources were mixed with glycerol to obtain a C/N ratio of 25 or 35. The lipid content (percent of CDW) was estimated using Bodipy fluorescence and the green fluorescent protein optical filter (ex: 486 nm/em: 508 nm; Gain = 80). Medium components are described below the figure and the concentration in g L^−1^ of each component is indicated after the corresponding letter (*YNB* yeast nitrogen base; *Nc* ammonium chloride; *Ns* ammonium sulfate; *G* glycerol)

For multiple species and strains of oleaginous yeasts, nitrogen sources are a critical factor for lipid accumulation [35, 36]. Because rich medium contains multiple sources of nitrogen (e.g., peptones, amino acids, ammonium sulfate, etc.), it complicates attempts to evaluate the effects of a given source. To better characterize the impact of nitrogen sources on lipid accumulation, then, we used a minimal defined medium (YNB medium) that contained one of two different nitrogen sources (ammonium sulfate or ammonium chloride) (Fig. 3). Regardless of the nitrogen source used, all strains reached the same biomass (13.4 ± 0.6 g L^−1^) and the same growth rates (0.32 ± 0.02 h^−1^), and no decrease in biomass was observed (data not shown). However, medium that contained ammonium chloride showed a higher potential to promote lipid accumulation than medium containing ammonium sulfate did at a given C/N ratio (Fig. 3). Similar results have already been reported for *Y. lipolytica* [44] and for the oleaginous yeast *Lipomyces starkeyi* [45]. As we found here, in both prior studies, a C/N of 35 led to higher accumulation of intracellular lipids.

### Impact of the carbon concentration at a constant carbon-to-nitrogen ratio

**Fig. 4:**
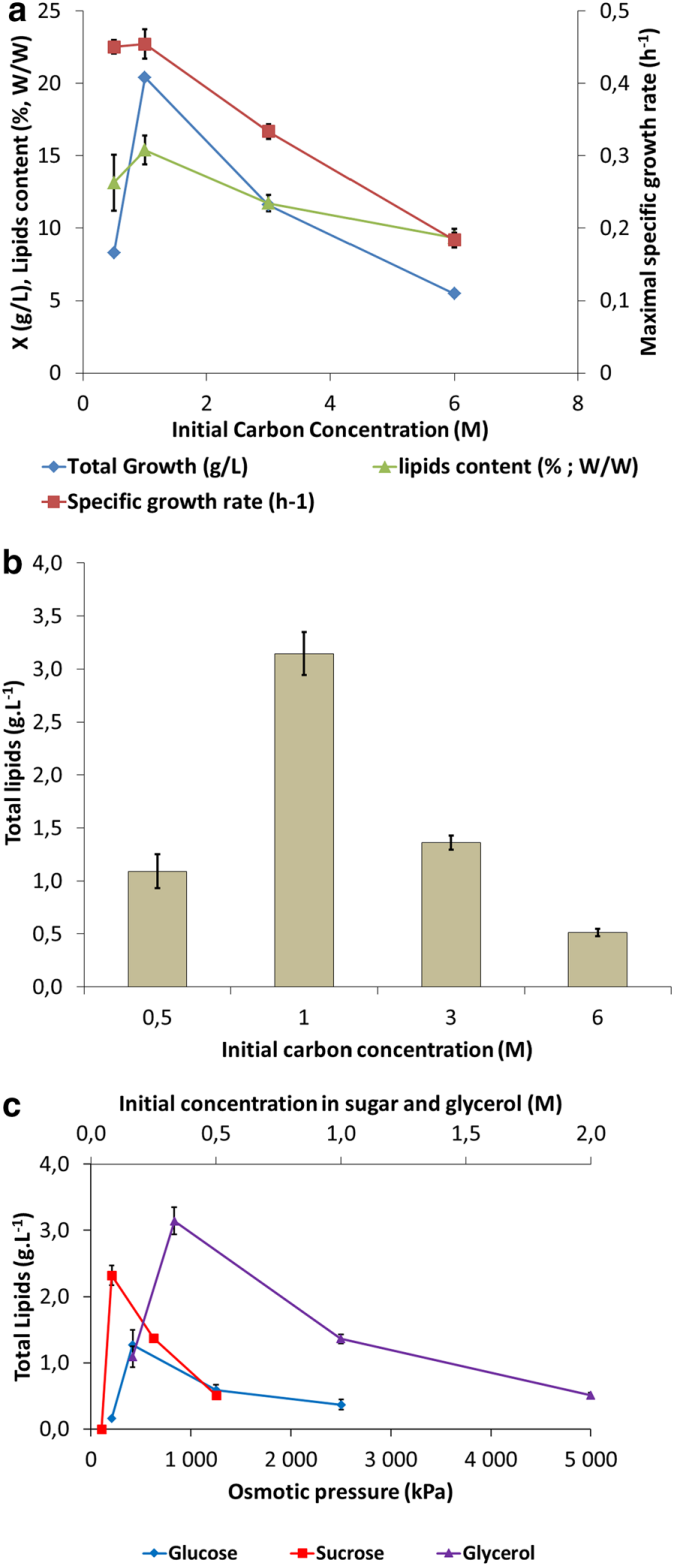
Impact of carbon concentration on *Y. lipolytica* JMY3675 growth at a constant C/N. The impact of the concentration of glycerol on the **a** biomass, specific growth rate, lipid content, and **b** total lipid concentration of strain JMY3675 grown on synthetic medium with a C/N ratio of 35. **c** The effect of the osmotic pressure (kPa) of glycerol, glucose, or sucrose on the production of total lipids. Osmotic pressure was calculated using a van’t Hoff factor of 1

The source and concentration of carbon are important parameters for the growth of a strain and its accumulation of intracellular lipids, as shown in Fig. 2. To evaluate the influence of carbon concentration, the growth of *Y. lipolytica* JMY3675 was monitored at different glycerol concentrations with a constant C/N ratio of 35. Under these conditions, biomass and specific growth rate were maximal at a carbon concentration of 1 M (30.6 g L^−1^ of glycerol) and quickly decreased with higher concentrations (i.e. 3 and 6 M) (Fig. 4a). The same effect was observed with regard to lipid accumulation and total lipid production (Fig. 4a, b). Similar effects were observed when glucose or sucrose was used as a carbon source (data not shown). This pattern could be explained by changes in osmotic pressure due to different concentrations of sugar or glycerol, which could then affect growth and lipid accumulation. This hypothesis has been proposed for multiple oleaginous yeasts, such as *Rhodosporidium toruloides* Y4 [46], *Cryptococcus curvatus* [47, 48], *Metschnikowia pulcherrima*, and *Y. lipolytica* [49]. To evaluate this, we investigated the effect of osmotic pressure on the production of lipids. The initial osmotic pressures of glucose, sucrose, or glycerol in the medium were calculated using the formula of van’t Hoff, where *i* is the van’t Hoff factor, C is the molar concentration, R is the gas constant (8.316 L kPa mol^−1^ K^−1^), and T is the temperature in Kelvin:

$$Osmotic \, Pressure \, = \, iCRT$$

We found that lipid production was negatively affected by osmotic pressures higher than 209 kPa in sucrose medium, 834 kPa in glycerol medium, and 417 kPa in glucose medium (Fig. 4c). All these pressures corresponded to 1 M carbon, which provided evidence that this carbon concentration was optimal for the production of lipids.

### Strain screening in microbioreactor for lipid production

Because the design of the BioLector is based on that of microtiter plates, it can quickly and easily be used to screen the culture conditions and strains that maximize production yields [20, 26]. In addition, previous works have demonstrated the efficiency of the BioLector in scaling up directly from microtiter plates to a bioreactor [21, 22]. We therefore used the BioLector to screen six strains of *Y.* *lipolytica* that differed in lipid accumulation in order to set up and validate a method to discriminate among strains based on lipid production traits. For this, we used the strains JMY2900, JMY1631, JMY3400, JMY3357, JMY3580, and JMY3675, and grew them in the culture conditions which had been determined to be optimal for JMY3675. The first strain, JMY2900, is a wild-type strain for lipid accumulation. Strain JMY1631 is derived from JMY2900, and lacks all *DGAT*s [17]. Because of this, this strain is unable to accumulate lipids into TAG and does not produce lipid bodies. Strains JMY3400, JMY3357, and JMY3580 are derived from strain JMY1631, and contain one, two, and four copies of the *DGA2* gene, respectively, which are expressed under the control of pTEF. Strain JMY3675 was included as a repeatability control. The complete genotype of these strains is described in Table 1.

**Fig. 5:**
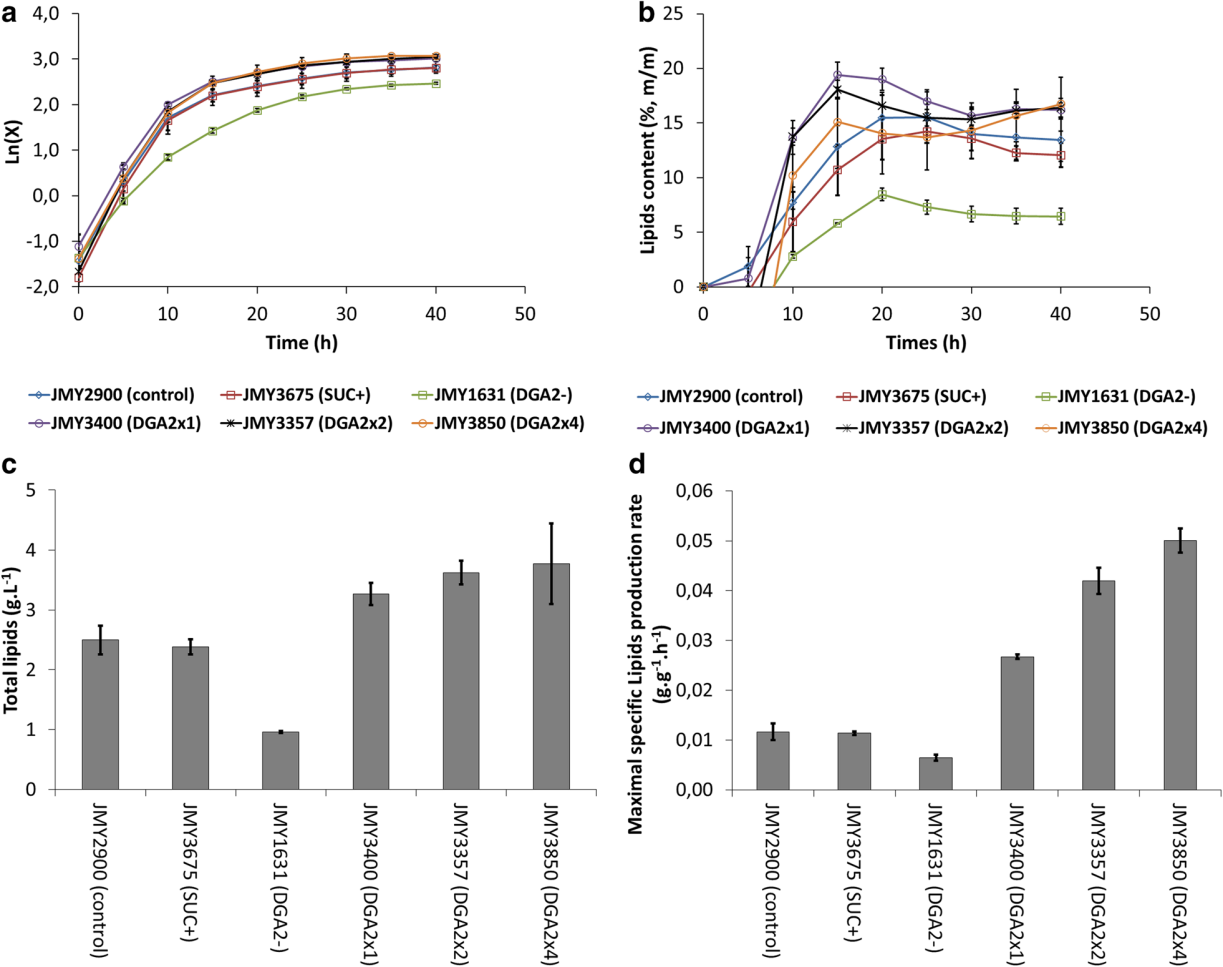
Growth and lipid accumulation by *Y. lipolytica* strains in optimized conditions. JMY2900 (control), JMY3675 (SUC^+^), JMY1631 (DGA^−^), JMY3400 (DGA2 × 1), JMY3357 (DGA2 × 2), and JMY3850 (DGA2 × 4) were cultured in YNB0.85Nc1.5G30.6 medium at a C/N ratio of 35. Growth was monitored in a 48-well flowerplate (filling volume: 800 µL, temperature: 28 °C, agitation: 1200 rpm) by measuring scattered light (ex: 620 nm/em: -, Gain: 10), and total lipids were monitored using Bodipy and the GFP optical filter (ex: 486 nm/em: 508 nm; Gain = 80). **a** was plotted by transforming units of scattered light into CDW (X in g L^−1^;* line*) and X in Ln(X). **b** The lipid yield was evaluated using Bodipy fluorescence and biomass (CDW). **c** Total lipid content and **d** maximal specific lipid production rate of the yeast strains

During growth in the BioLector, the specific growth rates of strains JMY2900, JMY3675, JMY3400, JMY3357, and JMY3580 were 0.275 ± 0.010 h^−1^, 0.298 ± 0.003 h^−1^, 0.298 ± 0.001 h^−1^, 0.315 ± 0.002 h^−1^ and 0.266 ± 0.007 h^−1^, respectively. Final biomass was very similar among these five strains, with 19.0 ± 1.7 g L^−1^, 19.3 ± 1.3 g L^−1^, 20.7 ± 0.4 g L^−1^, 20.9 ± 0.1 g L^−1^, and 21.4 ± 0.6 g L^−1^, respectively. Instead, the specific growth rate of JMY1631 appeared to be slower (0.176 ± 0.004 h^−1^) and its biomass lower (15.1 ± 0.7 g L^−1^), likely because it lacks *DGAT* genes and is unable to store TAG. In addition, a low fluorescence signal was detected for this strain due to elevated intracellular free fatty acid content [17]. This signal could be considered a baseline for lipid quantification in our conditions (Fig. 5a). As expected, the strains that expressed one or more copies of *DGA2* under the control of pTEF were more efficient in accumulating and producing lipids than the other strains (Fig. 5b, c). The maximal specific lipid production rate of each strain was determined using the following formula, where Qp is the maximal specific production rate (g g^−1^ h^−1^), P is the total lipid concentration (g L^−1^), t the time (h), and X the biomass (g L^−1^):

$$Qp\; = \;\frac{{d[P]}}{{dt}}\;\frac{1}{{[X]}}$$

Strains JMY2900 and JMY3675 had a maximal specific production rate of 0.012 ± 0.002 g g^−1^ h^−1^ and 0.011 ± 0.001 g g^−1^ h^−1^, respectively. The best maximal production rates were obtained with strains JMY3400 (0.027 ± 0.001 g g^−1^ h^−1^), JMY3357 (0.042 ± 0.003 g g^−1^ h^−1^), and JMY3580 (0.050 ± 0.002 g g^−1^ h^−1^), while the lowest (0.006 ± 0.001 g g^−1^ h^−1^) was observed with strain JMY1631 (Fig. 5d). Interestingly, the maximal specific production rate of intracellular lipids notably increased with the number of copies of *DGA2*, while lipid accumulation and lipid concentration remained in the same order of magnitude, increasingly only slightly (Fig. 5b–d). In this, our results were consistent with previous studies of the effect of *DGA2* on lipid production [17, 18].

### Scale up to bioreactor

Strains JMY2900, JMY3400, JMY3357, and JMY3580 were grown in a 500 mL stirred tank bioreactor for 48 h in YNB0.85Nc1.5G30.6 medium, and the results were compared from those obtained with the BioLector (Table 2). A higher final biomass was reached with the BioLector compared to the bioreactor cultures for all strains. However, the bioreactor yielded higher lipid content than the BioLector did. This could be because the flowerplate of the BioLector is designed to obtain a high oxygen transfer rate [50], and it has been demonstrated that higher amounts of dissolved oxygen increase citric acid production at the expensive of lipid synthesis [43, 44]. Overall, the lipid content determined during the comparison of the two fermentation instruments allowed the same strains to be discriminated for lipid accumulation. Recently, the BioLector showed its successful scale up or scale down from a microtiter plate to a stirred tank bioreactor for protein production [22, 46] and our results confirm that the BioLector is a useful tool for strain and medium screening prior to performing scale up in bioreactor. Even though there were differences between the two methods with regard to lipid accumulation, the fact that we were able to successfully cultivate the same strains using both approaches provides evidence of the BioLector’s utility in the scale-up process. The advantages that we have demonstrated that this apparatus provides with respect to a bioreactor—namely, the ability to rapidly screen a large number of strains and culture conditions—will enable time, money, and effort to be saved in the investigation of strains for industrial lipid production.

**Table 2:** Scale-up from BioLector to bioreactor

Strain	Biomass (g L^−1^)		Lipids content (%, W/W)	
	Bioreactor^a^	BioLector^b^	Bioreactor^c^	BioLector^c^
*Y. lipolytica* JMY2900	11.9	19.0 ± 1.7	9	10
*Y. lipolytica* JMY3400	16.0	20.7 ± 0.4	15	11
*Y. lipolytica* JMY3357	15.8	20.9 ± 0.1	17	14
*Y. lipolytica* JMY3580	13.4	21.4 ± 0.6	17	15

^a^ CDW was determined gravimetrically

^b^ Evaluated using scattered light

^c^ Quantified by gas chromatography

## Conclusion

Typically, microtiter plate readers are used to determine the optimal conditions for microbial growth and lipid production because they enable many strains and culture conditions to be screened simultaneously [20–22, 24, 47]. To this, the BioLector adds the ability to perform fermentation in conditions close to those of bench-top bioreactors, and, in addition, enables online fluorescence readings [21–23, 46]. To our knowledge, microfermentation systems like the BioLector have never before been used in studies of the model oleaginous yeast *Y.* *lipolytica*, either for real-time measurements of lipid production or for growth monitoring. Here, we demonstrated that this apparatus can be used to evaluate lipid accumulation in *Y. lipolytica* in real time using the fluorescence of a probe specific to intracellular lipids. We also took advantage of the high-throughput design of the BioLector to optimize growing conditions in order to increase lipid accumulation and production prior to performing larger-volume fermentations. The optimal culture conditions so determined were then applied to genetically modified yeast strains with different lipid-accumulation potencies, and fermentations were performed in parallel on the BioLector and in benchtop bioreactors. Higher biomass and lower lipid content were generated in the BioLector than in the bioreactors. In spite of this, the BioLector represents a useful tool for providing data about the conditions, media, and strains that are most suitable for scale-up. Because of this, the BioLector’s main advantage for the high-throughput fermentation screening of oleaginous yeasts is in facilitating time-consuming scale-up procedures. In addition, a recent development resulted in the BioLector being used for fed-batch cultures in microtiter plates, and this approach could be used to optimize lipid accumulation in *Y. lipolytica* [19]. The work presented here also demonstrates the role that carbon concentration plays in lipid production, showing that production was impaired at concentrations above 1 M. In the future, the fed-batch technique could be used to avoid the negative effect of high concentrations of carbon on the production of lipids.

## Abbreviations

C/N: carbon-to-nitrogen ratio
TAG: triacylglycerol
DAG: diacylglycerol
DGAT: DAG acyltransferase
YNB: yeast nitrogen base without amino acids and ammonium sulfate
Nc: ammonium chloride
Ns: ammonium sulfate
G: glycerol
Y: yeast extract
P: peptone
D: glucose
G: glycerol
S: sucrose
CDW: cell dry weight
GC: gas chromatography
*i*: Hoff factor
R: gas constant (8.316 L kPa mol^−1^ K^−1^)
Qp: maximal specific production rate (g g^−1^ h^−1^)
X: biomass (g L^−1^)
